# Toxin-Antitoxin Systems Alter Adaptation of Mycobacterium smegmatis to Environmental Stress

**DOI:** 10.1128/spectrum.02815-22

**Published:** 2022-11-01

**Authors:** Lan-Yue Zhang, Chun-Liang Wang, Mei-Yi Yan, Yi-Man Geng, Han Yin, Hong-Yan Jia, Chuan-Zhi Zhu, Zi-Hui Li, Gai-Xian Ren, Li-Ping Pan, Yi-Cheng Sun, Zong-De Zhang

**Affiliations:** a Beijing Key Laboratory for Drug-Resistant Tuberculosis Research, Beijing Chest Hospital, Capital Medical University, Beijing Tuberculosis and Thoracic Tumor Research Institute, Beijing, China; b MOH Key Laboratory of Systems Biology of Pathogens, Institute of Pathogen Biology, and Center for Tuberculosis Research, Chinese Academy of Medical Sciences and Peking Union Medical College, Beijing, China; c Clinical Laboratory Department, Henan Provincial People’s Hospital, People’s Hospital of Zhengzhou University, Zhengzhou, Henan, China; Shandong First Medical University

**Keywords:** *Mycobacterium smegmatis*, toxin-antitoxin system, bacteriophages, antiphage defense, iron-deficient environment

## Abstract

Toxin-antitoxin (TA) systems are ubiquitous genetic elements in prokaryotes, but their biological importance is poorly understood. Mycobacterium smegmatis contains eight putative TA systems. Previously, seven TAs have been studied, with five of them being verified as functional. Here, we show that Ms0251-0252 is a novel TA system in that expression of the toxin Ms0251 leads to growth inhibition that can be rescued by the antitoxin Ms0252. To investigate the functional roles of TA systems in M. smegmatis, we deleted the eight putative TA loci and assayed the mutants for resistance to various stresses. Deletion of all eight TA loci resulted in decreased survival under starvation conditions and altered fitness when exposed to environmental stresses. Furthermore, we showed that deletion of the eight TA loci decreased resistance to phage infection in Sauton medium compared with the results using 7H10 medium, suggesting that TA systems might have different contributions depending on the nutrient environment. Furthermore, we found that MazEF specifically played a dominant role in resistance to phage infection. Finally, transcriptome analysis revealed that MazEF overexpression led to differential expression of multiple genes, including those related to iron acquisition. Altogether, we demonstrate that TA systems coordinately function to allow M. smegmatis to adapt to changing environmental conditions.

**IMPORTANCE** Toxin-antitoxin (TA) systems are mechanisms for rapid adaptation of bacteria to environmental changes. Mycobacterium smegmatis, a model bacterium for studying Mycobacterium tuberculosis, encodes eight putative TA systems. Here, we constructed an M. smegmatis mutant with deletions of all eight TA-encoding genes and evaluated the resistance of these mutants to environmental stresses. Our results showed that different TA systems have overlapping and, in some cases, opposing functions in adaptation to various stresses. We suggest that complementary TA modules may function together to regulate the bacterial stress response, enabling adaptation to changing environments. Together, this study provides key insights into the roles of TA systems in resistance to various environmental stresses, drug tolerance, and defense against phage infection.

## INTRODUCTION

Toxin-antitoxin (TA) systems are widespread in prokaryotic genomes, including those of bacteria and archaea (1). TA modules have been initially classified into six types, types I to VI, based on the mechanism by which the antitoxin inactivates the toxin. A new type VII TA system was more recently described in which an enzymatic antitoxin chemically modifies the toxin posttranslationally to neutralize it (2). TA systems are involved in maintaining plasmid stability (3), bacterial persistence (4, 5), biofilm dynamics (6, 7), resistance to phage infection (8), adaptation to environmental stress (9), pathogenesis (10), and the regulation of gene expression (11–14).

The benefits of TAs to bacteria remain unclear, given that deletions of TA loci often have no effect on bacterial fitness. In addition, deletion of multiple TAs in Escherichia coli (15), Salmonella enterica (16), Staphylococcus aureus (17), and Pseudomonas putida (18) might not alter resistance to environmental stress or drug exposure. In contrast, several studies have also suggested that multiple TA systems synergize to alter bacterial stress tolerance. For example, deletions of MazF3, MazF6, and MazF9 RNase proteins impaired the survival of Mycobacterium tuberculosis exposed to oxidative stress and nutrient-limiting conditions, as well as in macrophage, guinea pig, and mouse infection models (10). The inability of Mycobacterium smegmatis to survive in complex medium following sequential deletion of three TA loci further illustrates that multiple TA systems may be required to adapt to complex environments (19).

M. tuberculosis, the etiological agent of tuberculosis (TB), is a major intracellular pathogen that encodes a large repertoire of at least 93 TA systems (20–22). Multiple TA systems may be expressed under different conditions, resulting in bacteriostasis and transcriptional reprogramming (10, 21, 23). Overexpression of the VapC22 system results in an altered transcriptome, comparable to that observed when M. tuberculosis is exposed to nutrient-limiting and low-oxygen conditions (23). Furthermore, overexpression of several toxins results in morphological changes and growth arrest, which may promote drug tolerance (24). It has also been reported that overexpression of VapC4 mimics amino acid starvation, activating oxidative and copper stress responses in M. tuberculosis (9). Despite multiple studies on the functions of TAs in M. tuberculosis, there remains a paucity of data on their role in resistance to environmental stresses (20, 22).

**FIG 1:**
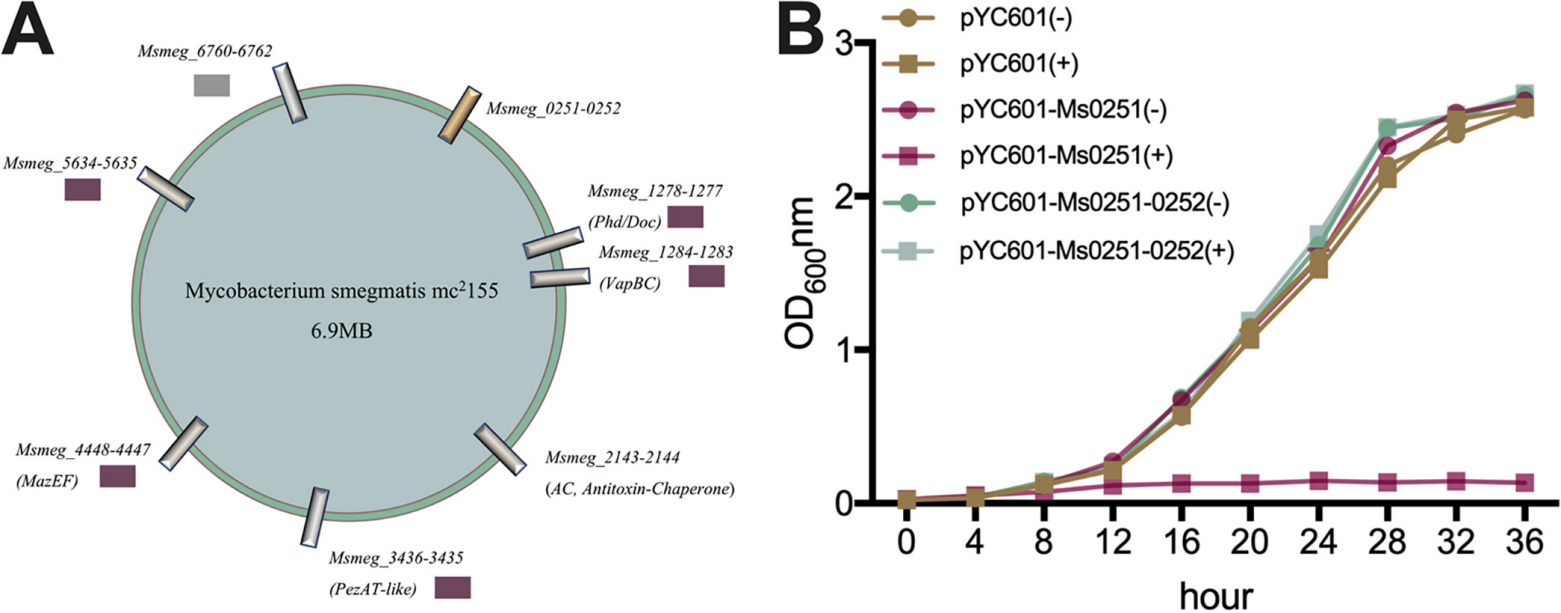
Toxin-antitoxin systems in M. smegmatis. (A) Genomic map of eight putative TA pairs in the genome of M. smegmatis mc^2^155. Purple squares indicate that functionality of the TA system has been experimentally confirmed. The gray square indicates that the toxin-antitoxin system is nonfunctional. (B) Growth curves of M. smegmatis with induced expression of different TA genes. The expression of *Ms0251*/*Ms0251-0252* in M. smegmatis was induced by the addition of 100 ng/mL ATc. The growth of each strain with or without the addition of ATc [(+) or (−)] was determined by measuring the absorbance at 600 nm (OD_600_) at regular intervals. The data shown in these panels are representative of three independent experiments.

We chose to use the nonpathogenic mycobacterium M. smegmatis due to its ease of manipulation and short generation time. The M. smegmatis mc^2^155 chromosome encodes eight putative TAs (Fig. 1A) (19, 20). Seven TAs have previously been studied in M. smegmatis, and five of them were confirmed as functional, including Ms1278-1277, Ms1284-1283, Ms3436-3435, Ms4448-4447 (MazEF), and Ms5634-5635 (25–28). In this study, we further demonstrated that Ms0252-0251 is also a functional TA system in M. smegmatis. Furthermore, we demonstrated that the deletion of all eight M. smegmatis TAs (Δ8TA strain) resulted in poor survival following nutrient starvation, increased resistance to H_2_O_2_ and isoniazid (INH), and decreased resistance to SDS, streptomycin, and phage infection. Finally, we showed that one TA module, MazEF, plays a dominant role in resistance to starvation, streptomycin exposure, and phage infection. In conclusion, this study provides new insights into the contribution of TAs to bacterial physiology in mycobacteria.

## RESULTS

### Experimental confirmation of putative TA systems.

Rv0207c-0208c, a homolog of Ms0251-0252, is a predicted TA system in M. tuberculosis (22). To verify whether *Ms0251-0252* is a functional TA system (Fig. 1A), the toxin-encoding gene, *Ms0251*, was cloned into an anhydrotetracycline (ATc)-based inducible vector and transformed into M. smegmatis. Induced expression of *Ms0251* inhibited the growth of M. smegmatis (Fig. 1B), and this inhibition could be rescued by coexpressing the antitoxin, *Ms0252*. These results show that Ms0251-0252 function as a TA pair, indicating that M. smegmatis encodes at least six functional TA modules (Fig. 1A).

### Phenotypic analysis of an eight-TA deletion mutant.

**FIG 2:**
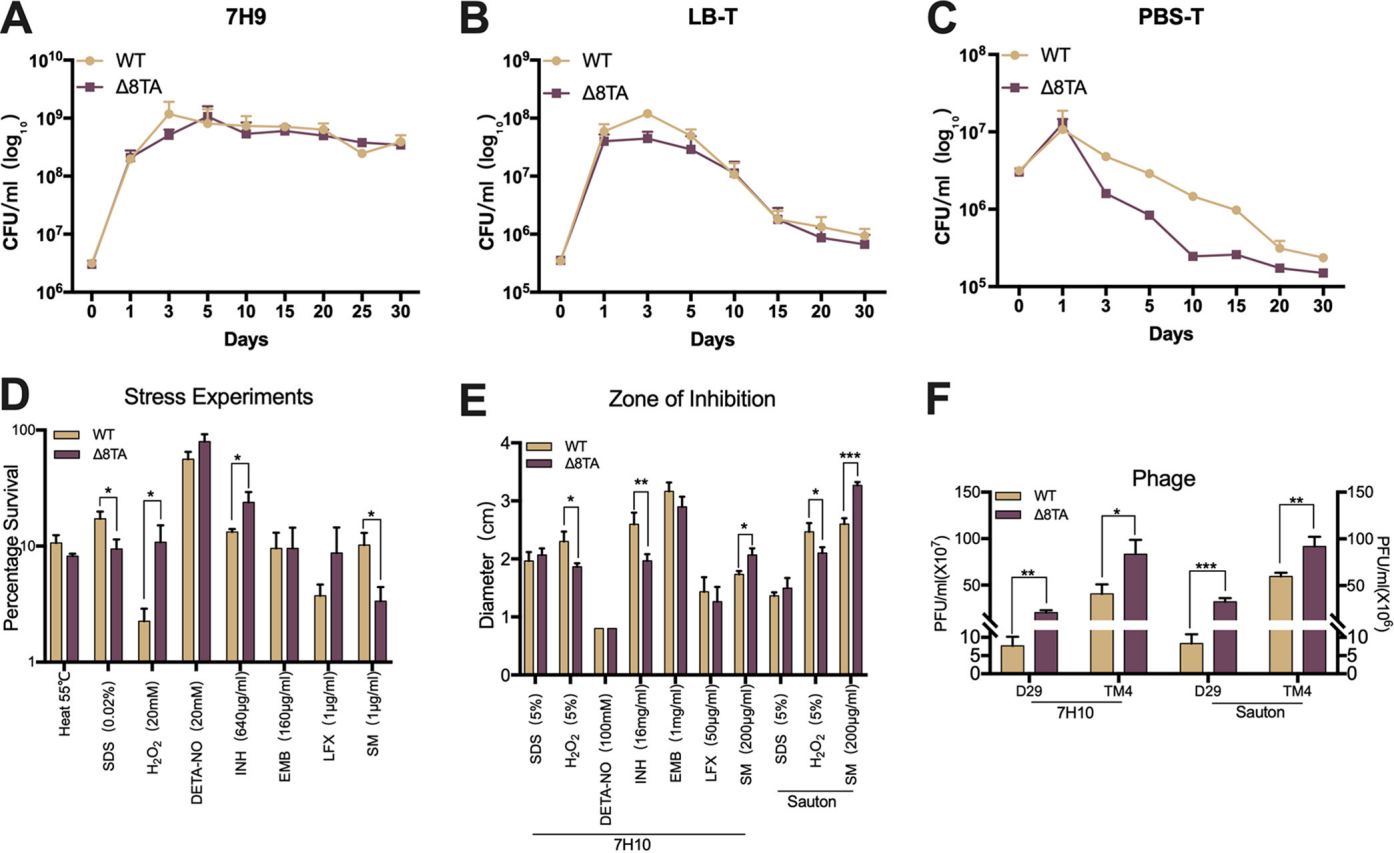
Susceptibility of the wild-type and Δ8TA strains to stress conditions. (A to C) Long-term nutritional stress was induced by incubation of bacteria in 7H9 (A), LB-T complex medium (B), and PBS-T minimal medium (C) for 30 days. CFU counts of the M. smegmatis wild-type and Δ8TA strains were monitored at regular intervals. (D) The wild-type and Δ8TA strains were grown in 7H9 medium to log phase before exposure to different stresses. Treatment concentrations and times are shown in the figure. Survival was monitored by plating serial 10-fold dilutions of culture on 7H10 agar. The percentages of survival were determined by comparing burdens of the stress exposure cultures to those of cultures prior to stress treatment. (E) Disc diffusion sensitivity assays of wild-type and Δ8TA strains following treatment with different stresses. Sensitivity was assayed in 7H10 or Sauton solid medium as measured by the zone of inhibition. (F) Plaque assays on 7H10 or Sauton solid medium, comparing the plaquing efficiency of D29 and TM4 phages on M. smegmatis wild-type and Δ8TA strains. Data from D29-exposed cultures are shown on the left *y* axis, while data from TM4-exposed cultures are shown on the right *y* axis. Results are the mean values ± standard deviations (SD) from three independent experiments. Statistically significant differences were determined using the paired two-tailed *t* test. *, *P < *0.05; **, *P < *0.01; ***, *P < *0.001.

To determine the fitness costs of multiple TA systems, the eight putative TA loci were sequentially deleted from the M. smegmatis mc^2^155 chromosome. Whole-genome sequencing analysis confirmed TA deletion and also revealed 15 point mutations in the Δ8TA strain, but none of these were present in genes known to affect persistence and growth. The Δ8TA mutant showed a growth profile in 7H9 medium that was comparable to the growth profile of the parental strain, indicating that the eight-TA deletion did not affect the growth of M. smegmatis (Fig. S1A in the supplemental material). TA modules are reported to contribute to long-time survival in M. smegmatis (19). To verify this observation, we monitored the numbers of CFU of the wild-type and Δ8TA strains during long-term incubation under aerobic conditions in 7H9 medium (Fig. 2A), LB supplemented with 0.05% Tween 80 (LB-T) (Fig. 2B), and PBS supplemented with 0.05% Tween 80 (PBS-T) (Fig. 2C). The Δ8TA and wild-type strains showed no significant differences in cell viability in 7H9 and LB-T complex media. Interestingly, a significant and highly reproducible survival defect was observed under aerobic conditions in PBS-T minimal medium (Fig. 2C). Together, these data demonstrated that deletion of the eight TA systems did not significantly affect the growth of M. smegmatis in 7H9 and LB-T complex media but did confer a fitness cost in PBS-T minimal medium.

TA systems are reported to affect the survival of bacteria during exposure to stresses, including heat shock, exposure to oxidative stress, surface stresses, nitrites, and drug treatment (12, 29, 30). We therefore evaluated whether TA modules were beneficial to M. smegmatis under different stress conditions. No significant differences between the Δ8TA mutant and the wild-type strain were observed following 55°C heat shock or exposure to 20 mM diethylenetriamine/nitric oxide adduct (DETA-NO), 160 μg/mL ethambutol (EMB), or 1 μg/mL levofloxacin (LFX) (Fig. 2D and Fig. S1D). In contrast, the Δ8TA strain was more susceptible to surface stress conferred by 0.02% SDS exposure and to treatment with 1 μg/mL streptomycin (Fig. 2D and Fig. S1D). Interestingly, the Δ8TA strain was more resistant to killing by 20 mM H_2_O_2_ oxidative stress and exposure to 640 μg/mL isoniazid (Fig. 2D and Fig. S1D). Consistent with the above-described results, disc diffusion antibiotic sensitivity assays showed that the Δ8TA strain was more sensitive to 200 μg/mL streptomycin and more tolerant of 5% H_2_O_2_ oxidative stress and 16 mg/mL isoniazid than the wild type in both 7H9 and Sauton medium (Fig. 2E and Fig. S1B).

TA systems are reported to play an important role in phage defense (8, 31–33). We therefore determined whether TA modules conferred resistance to phage infection by infecting the Δ8TA and wild-type strains with the mycobacterial phages D29 and TM4. The Δ8TA strain was slightly more susceptible than the wild-type strain to phage killing by D29 and TM4 in 7H10 medium (Fig. 2F and Fig. S1C), a phenotype which was greatly enhanced in Sauton solid medium. This medium-dependent difference in phage susceptibility may be explained by differential expression of TA systems in different culture conditions. Taken together, our results suggest that TAs play important and different roles in resistance to environmental stresses, drug exposure, and phage infection in M. smegmatis.

### TA-encoded proteins function divergently under different stress conditions.

**FIG 3:**
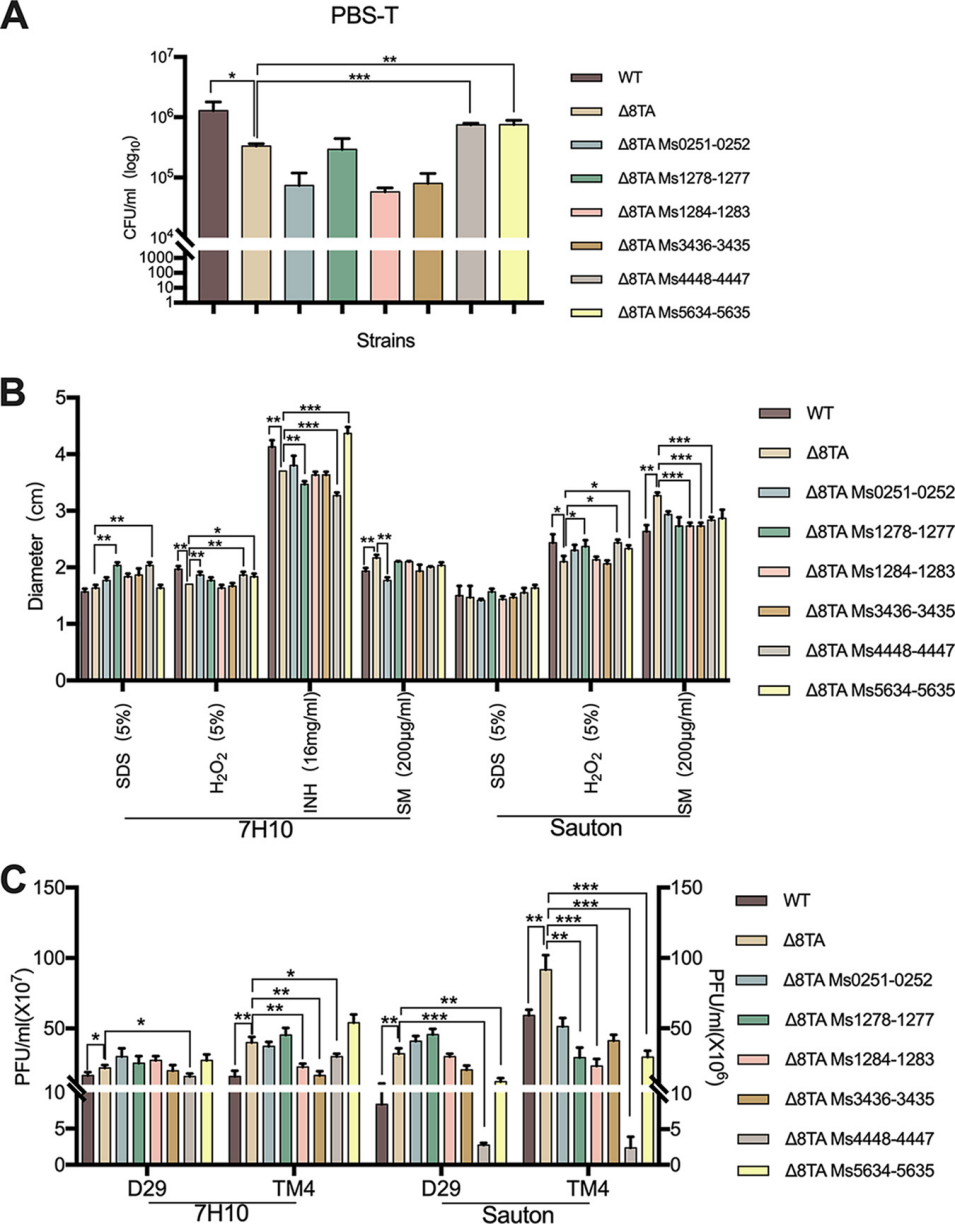
TA systems perform different functions following exposure to stress conditions. (A) M. smegmatis wild-type, Δ8TA, and ectopic-TA expression strains were grown in PBS-T medium for 10 days, and survival was monitored by plating of CFU. (B) Compound sensitivity as measured by the zone of inhibition in a disc diffusion assay using wild-type, Δ8TA, and ectopic-TA expression strains grown on 7H10 or Sauton solid medium. (C) Plaque assays on 7H10 or Sauton solid medium, comparing the plaquing efficiencies of D29 and TM4 phages on lawns of M. smegmatis wild-type, Δ8TA, and ectopic-TA expression strains. Data are the mean values ± SD obtained from three independent experiments. Statistically significant differences were determined using the paired two-tailed *t* test. *, *P < *0.05; **, *P < *0.01; ***, *P < *0.001.

To investigate which TA pairs in M. smegmatis function under different conditions, we expressed each individual functional TA pair with its endogenous promoter in the Δ8TA strain using a pMV261 vector, which has a low (3 to 5) copy number in M. smegmatis (34). The TA-expressing strains showed growth kinetics comparable to that of the empty vector control strain in 7H9 medium (Fig. S2A), indicating that ectopic expression of a single TA did not alter the proliferative activity of mycobacteria. The expression of Ms4448-4447 and Ms5634-5635 but not of the other TAs complemented the eight-TA mutant strain to wild-type (WT) levels of survival in PBS-T (Fig. 3A), indicating that these two TA modules play an important role in resistance to starvation.

The expression of Ms0251-0252 increased susceptibility to H_2_O_2_ and INH but increased resistance to streptomycin in 7H10 and Sauton solid medium. The expression of Ms1278-1277 or Ms4448-4447 decreased resistance to SDS and H_2_O_2_ treatment but increased resistance to INH treatment in 7H10 medium. In addition, the expression of any one TA system increased resistance to streptomycin in Sauton medium (Fig. 3B and Fig. S2B). Interestingly, the expression of Ms5634-5635 significantly decreased INH resistance (Fig. 3B and Fig. S2B), indicating that TA modules not only cause increased drug resistance but also decreased drug resistance.

Finally, we analyzed the role of TAs in resistance to phage infection. The expression of Ms4448-4447 slightly increased resistance to phage infection on 7H10 plates and greatly enhanced resistance to phage infection on Sauton medium plates. In addition, the expression of Ms5634-5635 slightly increased susceptibility to phage infection on 7H10 plates but increased resistance to phage infection on Sauton solid medium (Fig. 3C and Fig. S2C). Taken together, these results suggest that TA systems perform different functions following exposure to various stress conditions.

### Transcriptome analysis reveals a role of MazEF in iron metabolism.

**FIG 4:**
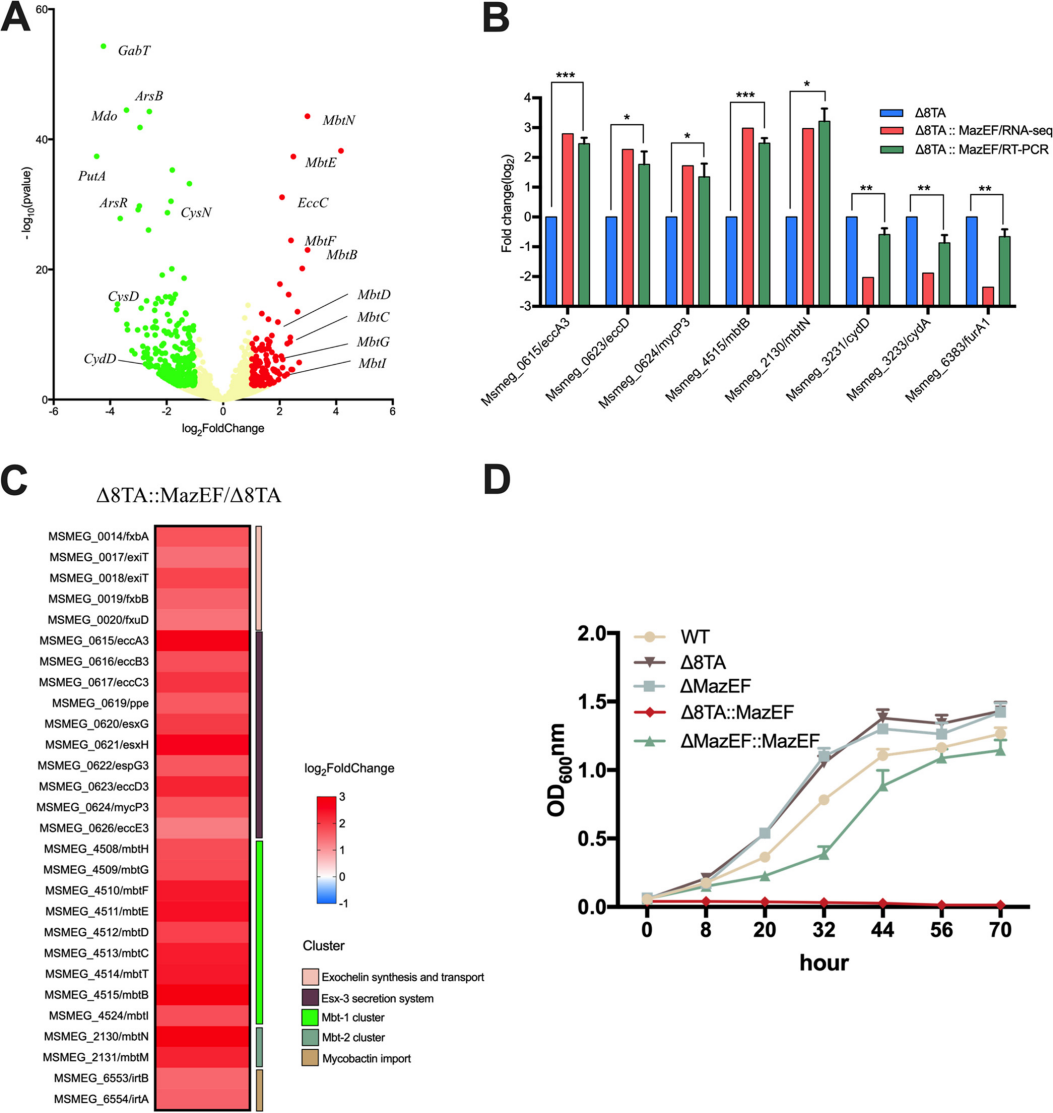
Effect of ectopic MazEF expression on the transcriptome of the M. smegmatis Δ8TA strain. (A) Volcano plot displaying differential gene expression profiles after expression of MazEF in the M. smegmatis Δ8TA strain. The *y* and *x* axes depict *P* value and fold change, respectively, for each gene. Statistically significant differentially expressed induced and repressed transcripts are highlighted as red and green dots, respectively. (B) Analysis of selected differentially expressed genes measured by RNA-seq and qRT-PCR in an MazEF overexpression strain compared with their values in the Δ8TA strain. qRT-PCR data were normalized to the expression of *sigA*, and the results are expressed as mean log_2_ fold change values ± SD from three independent biological replicates. (C) Heatmap showing log_2_ fold change in expression levels of iron-regulated genes following MazEF expression. Gene names and associated pathways are indicated. The data are representative of two biological replicates. (D) Growth curves of M. smegmatis derivative strains in Sauton fluid medium supplemented with 100 μM 2,2′-bipyridyl as determined by measuring the absorbance at 600 nm at regular intervals. The data are the mean values ± SD from three independent experiments. Statistically significant differences were determined using the paired two-tailed *t* test. *, *P < *0.05; **, *P < *0.01; ***, *P < *0.001.

MazEF (Ms4448-4447) plays an important role in resistance to starvation, streptomycin exposure, and phage infection. To determine the molecular mechanism underpinning the function of MazEF, we analyzed the transcriptome of the Δ8TA MazEF-expressing strain. We considered transcripts that were differentially expressed upon MazEF expression with an adjusted *P* value of <0.05 and a log_2_ fold change of ≤1.0 or ≥1.0 (Table S4). We found significantly different transcription of 408 genes following the expression of MazEF, with 129 being induced and 279 being repressed. This indicated that overexpression of MazEF alters the expression of ~10% of the M. smegmatis genome (Fig. 4A). The differential expression of five highly upregulated and three downregulated genes was further validated by real-time quantitative PCR (qRT-PCR), which confirmed changes in mRNA levels comparable to those seen by RNA sequencing (RNA-seq) (Fig. 4B).

We next annotated these differentially expressed genes according to their function category provided by Mycobrowser (https://mycobrowser.epfl.ch/). We found the *Mbt* gene cluster among those genes that were 4.0-fold upregulated following MazEF expression (Fig. S3). These genes encode key components of the “biosynthesis of siderophore group non-ribosomal peptides” pathway, which is responsible for the synthesis of iron-chelating siderophores (35). Furthermore, genes involved in iron acquisition, transport, and storage, in addition to other iron-regulated genes, were upregulated in the presence of MazEF (Fig. 4C). To further characterize the role of MazEF in iron acquisition, we assayed the growth of M. smegmatis strains in an iron-limited environment (Fig. 4D). MazEF strongly repressed the growth of M. smegmatis in an iron-limited environment but did not affect bacterial growth in iron-rich medium. These results suggest that MazEF regulates iron metabolism in M. smegmatis.

## DISCUSSION

Chromosomal TA systems are thought to act as stress response elements and contribute to bacterial stress resistance. This phenomenon relies on the ability of the toxin to inhibit bacterial growth when overexpressed or following deletion of the chromosomal antitoxin gene. Despite the potential of TA-encoded toxins to protect bacteria, the deletion of multiple TA loci does not significantly alter bacterial fitness (7, 36–39). M. tuberculosis encodes a large number of TA modules, which are predicted to play important roles in persistence (5), stress adaptation (9), and pathogenesis (10). In this study, we systematically characterized TA systems from M. smegmatis, a model organism for the study of M. tuberculosis, and identified a new functional TA module, Ms0251-0252 (Fig. 1).

Deletion of the eight TAs in M. smegmatis resulted in decreased survival under starvation stress when grown in PBS-T (Fig. 2C) but not when grown in 7H9 rich medium and LB-T complex medium for 30 days (Fig. 2A and B). Our results are contrary to previous reports that the deletion of three TAs in M. smegmatis (3TA mutant) caused no survival after 15 days of growth in LB-T complex medium (19). One reason for this disparity is the potential for mutations between wild-type M. smegmatis strains in different laboratories due to serial passage. Another possible reason is the presence of an unexpected mutation in the 3TA mutant, which had not been sequenced. We further demonstrated that the deletion of eight TAs in M. smegmatis altered its ability to survive in different stress environments. Compared with the wild-type strain, the Δ8TA mutant had different susceptibilities to treatment with SDS, streptomycin, H_2_O_2_, and isoniazid and to phage infection (Fig. 2D to F).

Different TA modules might play different or even competing roles in adaptation to various environmental stresses. Ectopic expression of Ms1278-1277 or Ms4448-4447 resulted in increased resistance to isoniazid, but the expression of Ms0251-0252 or Ms5634-5635 resulted in decreased resistance to isoniazid (Fig. 3B). TAs may be differentially expressed under specific conditions, promoting adaptation to changing environments. For example, MazEF only slightly increased the resistance to phage infection when M. smegmatis was grown on 7H10 plates, but it strongly increased resistance to phage infection following growth on Sauton solid medium (Fig. 3C). We propose that TA modules coordinately regulate the stress response to promote adaptation to changing environments.

Our RNA-seq analysis revealed that the expression of MazEF mimics an iron-deficient environment and results in increased expression of genes involved in iron acquisition (Fig. 4C). The toxin MazE functions as an endoribonuclease to cleave a single site within the anticodon sequences (UU↓U and CU↓U) of the target protein, leading to ribosome stalling at Lys AAA codons. Cleavage results in selective degradation of mRNAs harboring ribosomes stalled at Lys codons, remodeling the bacterial proteome (40). Proteomics analysis showed that the expression of MazE alters the expression of multiple proteins but does not significantly alter the expression of proteins involved in iron acquisition (40). This may be explained by discordance between mRNA levels and the expression of protein products (41, 42). These data provide impetus to study the biological roles of MazEF expression.

Taken together, this is the first study to systematically decipher the role of all TA systems in M. smegmatis, which may provide useful insights into the roles of TAs in resistance to various environmental stresses, drug tolerance, and defense against phage infection in M. tuberculosis. The data presented in this study indicate that different TA modules might play different roles and coordinately function to allow M. smegmatis to adapt to changing environmental conditions. Furthermore, the eight-TA deletion mutant generated in this study may provide a molecular framework to study the functions of TAs in M. tuberculosis.

## MATERIALS AND METHODS

### Bacterial strains and culture conditions.

Escherichia coli strain DH5α, M. smegmatis strain mc^2^155, and their derivatives were used in this study. E. coli cells were grown in Luria-Bertani (LB) medium supplemented with appropriate antibiotics. M. smegmatis was grown in Middlebrook 7H9 broth (Difco) supplemented with 0.05% Tween 80 and 0.2% glycerol or on Middlebrook 7H10 agar supplemented with the appropriate antibiotic. The growth medium was supplemented with 25 μg/mL kanamycin, 100 μg/mL ampicillin, 50 μg/mL zeocin, 50 μg/mL hygromycin, or 50 ng/mL anhydrotetracycline (ATc) depending on the required selection.

For starvation or stress experiments, M. smegmatis was grown in LB, phosphate-buffered saline (PBS), or Sauton medium (SM) (4 g/L asparagine, 0.5 g/L KH_2_PO_4_, 2.2 g/L citric acid monohydrate, 1 g/L MgSO_4_·7H_2_O, 60 mL/L glycerol, adjusted to pH 7.4 with NaOH), all supplemented with 0.05% Tween 80. Unless otherwise stated, both E. coli and M. smegmatis were cultured at 37°C.

To verify the function of TA systems, cultures were induced with 100 ng/mL ATc and growth assays were performed in 7H9 medium with shaking at 200 rpm. For long-term mild nutrient starvation experiments (19), early-log phase cultures (optical density at 600 nm [OD_600_] of ~0.3) were washed twice with PBS and resuspended in 7H9 and PBS-T media for 30 days. Mid-log-phase cultures (OD_600_ of ~0.8) were passaged once in LB-T to an initial OD_600_ of ~0.02 and resuspended in LB-T medium for 30 days before monitoring bacterial cell viability by counting CFU.

### Construction of plasmids and strains.

The genes encoding TA systems were amplified from the M. smegmatis chromosome for recombinant-plasmid construction. The M. smegmatis Δ8TA strain was constructed by sequential deletion of its eight TA loci from M. smegmatis using pCR-sgRNA plasmids that encoded either hygromycin or zeocin resistance and contained TA system genes (43). The ΔMazEF strain was constructed using the same protocol. The inducible-expression plasmids were constructed by cloning TA genes into pYC601. The complementation plasmids were constructed by cloning TA genes, including 500 bp of upstream and downstream sequences, into pMV261. The bacterial strains and plasmids used are listed in Table S1. The primers used in PCR amplification are listed in Table S2.

### Whole-genome sequencing.

The Δ8TA strain and WT M. smegmatis strain mc^2^155 were incubated at 37°C until the OD_600_ reached 0.6 to 0.8. Chromosomal DNA was extracted as previously described (44) before sonication into fragments shorter than 500 bp. The fragments were treated with end prep enzyme mix for end repair, 5′ phosphorylation, and dA tailing in one reaction, followed by a T-A ligation to add adaptors to both ends. Size selection of adaptor-ligated DNA was performed, and fragments of ~470 bp were recovered. Each sample was then amplified by PCR for eight cycles using P5 and P7 primers. The PCR products were purified and validated using an Agilent 2100 Bioanalyzer and quantified using a Qubit 3.0 fluorometer (Invitrogen, Carlsbad, CA, USA). Libraries with different indices were multiplexed and loaded on an Illumina HiSeq or NovaSeq (Illumina, San Diego, CA, USA) according to the manufacturer’s instructions. Sequencing was carried out using a 2 × 150-bp paired-end-read configuration, generating approximately 500-fold coverage of the genome.

### Persistence assays.

To quantify persistence after antibiotic treatment, bacteria were grown overnight in 4 mL of 7H9 medium. The overnight-grown cultures were diluted 64-fold into 7H9 medium and grown at 37°C to exponential growth phase (OD_600_ of ~0.7). Bacteria were subjected to heat shock at 55°C or treated with 0.02% SDS, 20 mM H_2_O_2_, or 20 mM DETA-NO for 2 h with shaking at 37°C. Bacteria were treated with isoniazid (INH), ethambutol (EMB), levofloxacin (LFX), and streptomycin (SM) for 20 h. Isoniazid was used at 640 μg/mL, ethambutol at 160 μg/mL, and levofloxacin and streptomycin at 1 μg/mL. One milliliter of bacterial culture was centrifuged and resuspended in phosphate buffered saline (PBS), and 10-fold serial dilutions of the cultures were spotted as 5-μL drops onto 7H10 plates before incubation at 37°C for 48 to 72 h. We used the compound concentration with a survival rate of about 10% after 2 h of treatment or the drug concentration with a survival rate of 10% after 20 h of treatment.

### Disk diffusion assays.

The sensitivity of M. smegmatis mc^2^155 strains to different antibiotic or compound treatments was determined using a disk diffusion assay. M. smegmatis mc^2^155 wild-type, Δ8TA, and recombinant-mutant strains were grown overnight in 7H9 broth before dilution to an OD_600_ of ~0.7. Five hundred microliters of bacterial culture was mixed with 2,500 μL of 7H9 broth and 3 mL of 7H9 soft agar before plating onto 7H10 agar plates containing the appropriate antibiotic(s). Sterile filter paper disks saturated with 10 μL of SDS (5%), H_2_O_2_ (5%), DETA-NO (100 mM), isoniazid (INH) (16 mg/mL), ethambutol (EMB) (1 mg/mL), levofloxacin (LVX) (50 μg/mL), or streptomycin (200 μg/mL) were placed onto the hardened agar, and the plates were incubated at 37°C for 48 h. Sauton fluid medium was used for determination of sensitivity in limiting medium. We used the compound or drug concentration that resulted in a 1- to 4-cm-diameter zone of inhibition after 48 h of treatment. The diameters of the zones of growth inhibition were recorded and analyzed statistically.

### Plaque assays.

Phage lysates were 10-fold serially diluted, and 5-μL amounts were spotted onto top agar overlays containing 0.5 mL of M. smegmatis mc^2^155 or its derived strains using Middlebrook 7H10 with 0.35% agar and 1 mM CaCl_2_. Plates were incubated at 37°C for 24 to 48 h until visible plaques formed before quantifying the PFU (45). Sauton solid medium was used for determination of sensitivity in limiting medium.

### RNA isolation and qRT-PCR.

RNA was extracted according to the FastRNA pro blue kit manufacturer’s instructions. Briefly, 3 mL of bacteria (OD_600_ of ~1.0) was resuspended in RNA*pro* solution. Bacterial suspensions were processed in the FastRNA instrument (MP FastPrep system; MP Biomedicals) for 40 s at a setting of 6.0. The lysed cell mixture was extracted with chloroform, and the RNA pellet was dissolved in RNase-free water. A total of 200 ng of purified RNA was reverse transcribed to cDNA using HiScript II reverse transcriptase (Vazyme, Ltd.) according to the manufacturer’s protocol. qRT-PCRs were performed using PowerUP SYBR green master mix (Applied Biosystems, Inc.) in the ABI 7900 real-time PCR system (Applied Biosystems, Inc.). Data were normalized to *sigA* gene expression levels. The cycle threshold (2^−ΔΔ^*^CT^*) method was used to analyze relative changes in gene expression.

### RNA sequencing and analysis.

Total RNA was extracted using the TRIzol reagent/RNeasy minikit (Qiagen). rRNA was depleted from total RNA using the rRNA removal kit. The depleted rRNA was then fragmented and reverse transcribed. The first cDNA strand was synthesized using ProtoScript II reverse transcriptase with random primers and actinomycin D. The second cDNA strand was synthesized using the second-strand synthesis enzyme mix. dA tailing was performed in one reaction, followed by a T-A ligation to add adaptors to both ends. Size selection of adaptor-ligated DNA was then performed using beads, and fragments of ~400 bp were recovered. Each sample was then amplified by PCR using the P5 and P7 primers. The PCR products were validated using a Qsep100 (Bioptic, Taiwan, China) and quantified using the Qubit 3.0 fluorometer. Libraries with different indices were multiplexed and loaded on an Illumina HiSeq/NovaSeq instrument according to the manufacturer’s instructions. Sequencing was carried out using a 2 × 150-bp paired-end-read configuration.

### Statistical analysis.

The data from this study were analyzed with the paired *t* test. Values of *P < *0.05 were defined as statistically significant. The differences between the three biological replicates in the experiment were expressed as error bars.

### Data availability.

Sequences of M. smegmatis mc^2^155 and the Δ8TA strain are available in NCBI under accession number PRJNA851567. RNA-seq data from Δ8TA and Δ8TA::MazEF strains are available in NCBI under accession number PRJNA852255.
