# Supplementary material for: Toxin-Antitoxin Systems Alter Adaptation of Mycobacterium smegmatis to Environmental Stress
Source: Microbiol Spectr. 2022 Nov 1;10(6):e02815-22. doi: 10.1128/spectrum.02815-22 (PMC9769933; doi:10.1128/spectrum.02815-22)
Supplement: Supplemental file 1 — Tables S1 to S3 and Fig. S1 to S3. Download spectrum.02815-22-s0001.pdf, PDF file, 4.4 MB [file spectrum.02815-22-s0001.pdf]

Supplementary Materials for Toxin-antitoxin systems alter adaptation of *Mycobacterium Smegmatis* to environmental stress

Table S1 Bacterial strains and plasmids used in this study.

	**Description**	**References**
**Bacterial strains**		
E.coli DH5α	cloned strain	
SY1918	Wild-type strain of *M. smegmatis* mc^2155	this study
SY6201	SY918 contains pYC601	this study
SY7853	SY918 contains pYC601-Ms0251	this study
SY7996	SY918 contains pYC601- Ms0251-0252	this study
SY3368	SY918 contains pMV261	this study
SY6941	Δ8TA mutant strain of *M. smegmatis*	this study
SY7439	SY6941 contains pMV261	this study
SY7440	SY6941 contains pMV261- Ms0251-0252	this study
SY7441	SY6941 contains pMV261- Ms1278-1277	this study
SY7442	SY6941 contains pMV261- Ms1284-1283	this study
SY7443	SY6941 contains pMV261- Ms3436-3435	this study
SY7444	SY6941 contains pMV261- Ms4448-4447	this study
SY7445	SY6941 contains pMV261-Ms5634-5635	this study
SY8609	SY6564 contains pMV261	this study

SY6472	Ms4448-4447 mutant strain of *M. smegmatis*	this study
SY7857	SY6472 contains pMV261-Ms4448-4447	this study
**Plasmids**		
pYC601	Tetracycline Inducible Expression Plasmid. (Hyg^r)	[1]
pCR-Hyg	Shuttle vector containing crRNA cassette which inserted *BpmI* and *HindIII* sites between Direct repeats downstream to Hsp60 promoter. (Hyg^r)	[1]
pCR-Zeo	Zeo replace hyg resistance in pCR-Hyg. (Zeo^r)	[1]
pJV53-Cas12a	Optimized Cpf1 under control of the $P_{myc1}tetO$ promoter inserted in pJV53. (Kn^r)	[1]
pMV261	Shuttle vector, replicates extrachomosomally in both *E.coli* and *Mycobacterium*. (Kn^r)	[2]
pYC2487	pYC601 harboring Ms0251 region	this study
pYC2424	pYC601 harboring Ms0251-0252 region	this study
pYC2425	pMV261 harboring Ms0251-0252along with the flanking region	this study
pYC2127	pMV261 harboring Ms1278-1277 along with the flanking region	this study
pYC2131	pMV261 harboring Ms1284-1283 along with the flanking region	this study
pYC2154	pMV261 harboring Ms3436-3435 along with the flanking region	this study
pYC2129	pMV261 harboring Ms4448-4447 along with the flanking region	this study
pYC2130	pMV261 harboring Ms5634-5635 along with the flanking region	this study

Table S2 Oligonucleotides used in this study.

Primer Name	Forward Primer (5'-3')	Reverse Primer (5'-3')
Ms0251-0252 crRNA	atttgctggaggtcctccacgatgtcga	agcttcgacatcgtggaggacctccagcaaatct
Ms1278-1277 crRNA	atggctgagctcaaccgccgcttggcga	agcttcgccaagcggcggttgagctcagccatct
Ms1284-1283 crRNA	atggactatgtccaccgcgtcgtatcta	agcttagatacgacgcggtggacatagtccatct
Ms2143-2144 crRNA	atgaaagtgggcgcgccgtcccgctcaa	agctttgagcgggacggcgcgcccactttcatct
Ms3436-3435 crRNA	atgtcgaactcacattggccccgctgca	agcttgcagcggggccaatgtgagttcgacatct
Ms4448-4447 crRNA	atgtcgacgcgatctccgtcgactggga	agcttcccagtcgacggagatcgcgtcgacatct
Ms5634-5635 crRNA	attccagggtctcgggcagcttcgagca	agcttgctcgaagctgcccgagaccctggaatct
Ms6760-6762 crRNA	atacatcggtcacgggcatgtcagtcca	agcttggactgacatgcccgtgaccgatgtatct
pYC601- Ms0251	tccgcatgcggaggaatcaggtgaacgcttcagca ggccc	gtccccaattaattagctaatcacacacgcgaggt cagta
pYC601- Ms0251-0252	tccgcatgcggaggaatcaggtgacgcccgacatc gtgga	gtccccaattaattagctaatcacacacgcgaggt cagta
pMV261- Ms0251-0252	taactacgtcgacatcgataggtgtcgggggccttg agcc	ccgtggcgcggccgcggtaccaccgccagctca cccggcc
Ms0251-0252	gtcgtcgtcaccttggagcg	ggtcctctggtttgagcacc
Ms1278-1277	tgtcaacgactgctgacgcc	gcagtgatcgcgtcgtaatc
Ms1284-1283	cagcagctctaccgggaact	cggcgtggtgatctgatcaa
Ms2143-2144	atcacatcggtgactgagcg	ggcggcgtgagtcttgaatt
Ms3436-3435	cgtccagttcaaccgagact	atttcggtttggtggtccact
Ms4448-4447	cacgcgtcgattcactgtcc	gtcttcacggtcgaaggact
Ms5634-5635	ttcaagcgccccaaacggta	aggaactgttcatgctggat
Ms6760-6762	taccgcgtgatggcaaagga	gatgttcggcaccaggttct
SigA-qPCR	cgagcttgttgatcacctcgaccat	ctcgacctcatccaggaaggcaac
Ms0615-qPCR	attcctcgacaccaacgaag	gtgaacagacgctccatgtc
Ms0623-qPCR	ttcgtgatcggcgatcggta	cagcgaataggtgtccggcg

Ms0624-qPCR	ggatcatcgcgttcgtgggtac	gtcttgtccttccgacggtagg
Ms4515-qPCR	gcgaccgacgaattccagtg	agaagttcgtcacctggtgc
Ms2130-qPCR	aagatgtgcatgcactccga	cgcgtcgacatgctgcgcta
Ms3231-qPCR	cgtgaaatacgggcgcagac	tgtcgcgcaatggttccagg
Ms3233-qPCR	aagatccacagcccgatgaacg	gaccggcatcgtgcaggaattc
Ms6383-qPCR	tgttgcggtccgtgtacgca	tagcgcgccaccgaacctga

Table S3 Whole-genome sequencing of *M. smegmatis* wild-type strain and Δ8TA strain.

POS IN WT	GENE	GENE NAME	REF-ALT	PRODUCT	MTB HOMOLOGOUS GENE
1013717	Ms0925		C-T	hypothetical protein	
1078425	intergenic region		A-G		
1316237	intergenic region		A-G		
3405540	intergenic region		A-G		
3573325	Ms3496	mmpL5	A-G	Siderophore exporter	Rv0676c
3845520	Ms3767		G-A	2-succinylbenzoate-CoA ligase	Rv1683
4290711	Ms4200		G-A	Succinyl-diaminopimelate desuccinylase	Rv2141c
5486992	Ms5393	kdpB	C-T	Potassium-transporting ATPase ATP-binding subunit	Rv1030
6105606	Ms6025		G-A	hypothetical protein	Rv1378c, Rv3074
6339524	Ms6258		C-A	hypothetical protein	
6406683	Ms6322	tgs2	C-T	putative diacylglycerol O-acyltransferase	Rv3734c
6444602	Ms6373		G-A	3-keto-5-aminohexanoate cleavage enzyme	
1042845	Ms0961		C-T		
1126687	Ms1052		C del	amino acid carrier protein	
6726802	Ms6655		A-G	hypothetical protein	

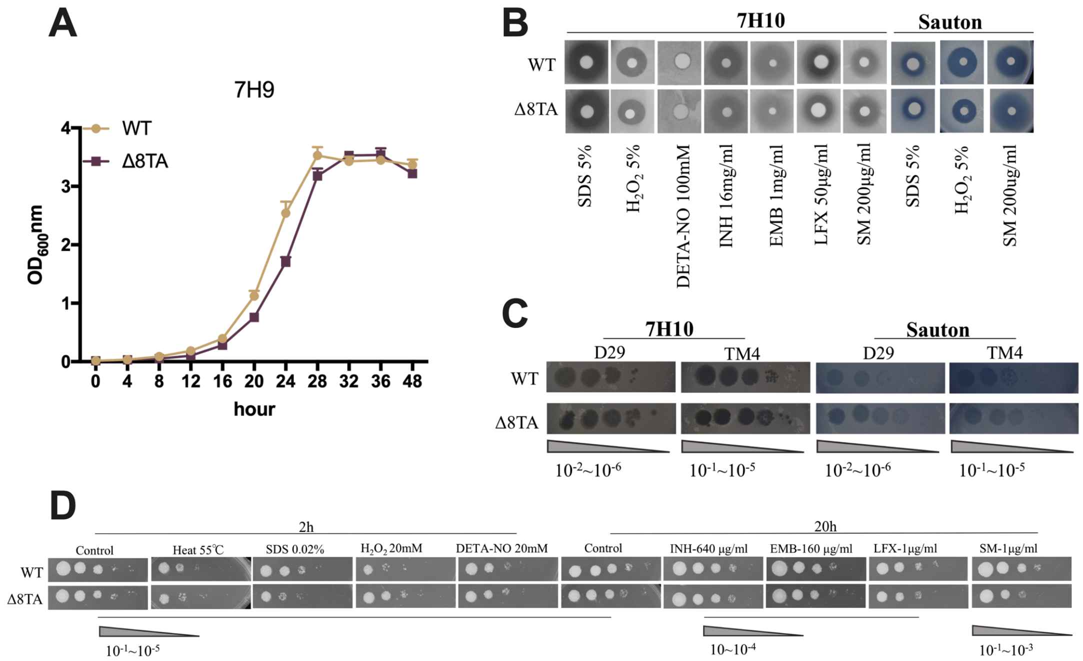

FIG S1 Susceptibility of the wild type and Δ8TA strains to stress conditions.

(A) Growth curves of of *M. smegmatis* wild type and Δ8TA strain were determined by measuring absorbance at 600 nm (OD_{600}) at regular intervals. (B) Disc diffusion sensitivity assays of wild type and Δ8TA strains following treatment with different stresses. Sensitivity was assayed in 7H10 or Sauton solid medium as measured by the zone of inhibition. (C) Plaque assays on 7H10 or Sauton solid medium, comparing the plaquing efficiency of D29 and TM4 phages on *M. smegmatis* wild type and Δ8TA strains. (D) The wild type and Δ8TA strains were grown in 7H9 medium to log phase, before exposure to different stresses. Treatment concentrations and times are shown in the figure. Data obtained from three independent experiments.

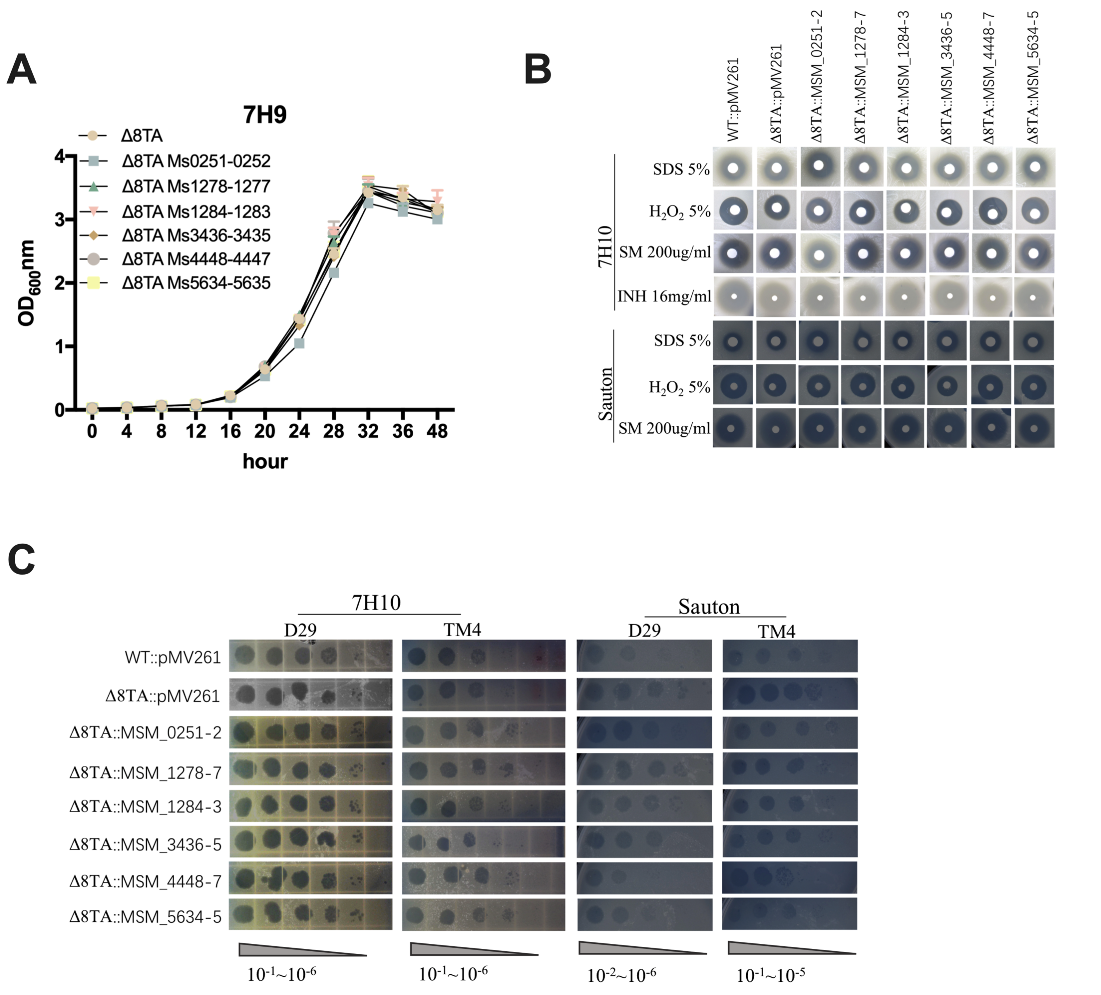

FIG S2 TA systems perform different functions following exposure to stress conditions**.** (A) Growth curves of *M. smegmatis* wild-type, Δ8TA and ectopic TA expression strains were determined by measuring absorbance at 600 nm (OD_{600}) at regular intervals. (B) Disc diffusion sensitivity assays of *M. smegmatis* wild-type, Δ8TA and ectopic TA expression strains following treatment with different stresses. Sensitivity was assayed in 7H10 or Sauton solid medium as measured by the zone of inhibition. (C) Plaque assays on 7H10 or Sauton solid medium, comparing the plaquing efficiency of D29 and TM4 phages on *M. smegmatis* wild-type, Δ8TA and ectopic TA expression strains. Data obtained from three independent experiments.

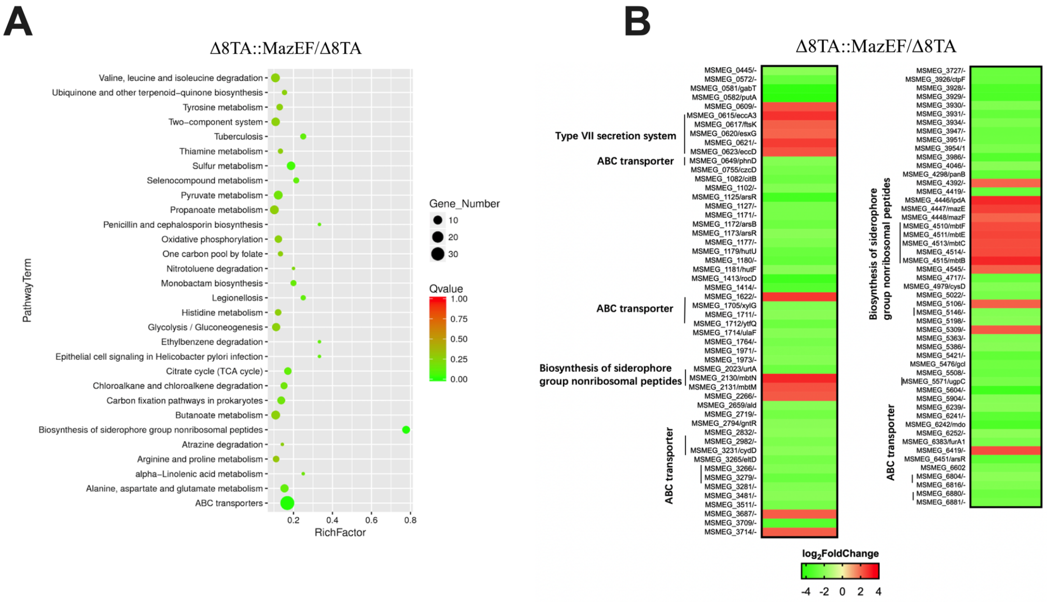

FIG S3 Effect of ectopic MazEF expression on the transcriptome of the *M. smegmatis* Δ8TA strain**.** (A) KEGG enrichment scatter plot of differential gene expression after expression of MazEF in the *M. smegmatis* Δ8TA strain. The *y* axis represents the name of the pathway, the *x* axis represents the Rich factor, the size of the point represents the number of differentially expressed genes in this pathway, and the color of the point corresponds to different Qvalue ranges. (B) Heatmap showing transcripts differentially expressed > 4.0-fold following MazEF expression. Gene names and associated pathways are indicated to the left of heat map. The data are representative of two biological replicates.

REFERENCES

1. XJ M, MY Y, H Z, XP G, YC S: **Efficient and simple generation of multiple unmarked gene deletions in Mycobacterium smegmatis**. *Scientific reports* 2016, **6**(undefined):22922.
2. CK S, VF dlC, TR F, JE B, LA B, LT B, GP B, JF Y, MH L, GF H: **New use of BCG for recombinant vaccines**. *Nature* 1991, **351**(6326):456-460.
